# Finite Element Modeling of Quantitative Ultrasound Analysis of the Surgical Margin of Breast Tumor

**DOI:** 10.3390/tomography8020047

**Published:** 2022-03-01

**Authors:** Koushik Paul, Samuel Razmi, Barbara A. Pockaj, Leila Ladani, Jeremy Stromer

**Affiliations:** 1 School for Engineering of Matter, Transport and Energy, Ira A. Fulton Schools of Engineering, Arizona State University, Tempe, AZ 85281, USA; leila.j.ladani@gmail.com; 2 EnMed Department, Texas A&M College of Medicine, Houston, TX 77807, USA; samrazmi@tamu.edu; 3 Mayo Clinic in Arizona, Surgery, Phoenix, AZ 85054, USA; pockaj.barbara@mayo.edu; 4 Survivability Engineering Branch, US Army Engineer Research and Development Center, Vicksburg, MS 39180, USA; jeremy.d.stromer@erdc.dren.mil

**Keywords:** quantitative ultrasound, finite element analysis, surgical margin, breast cancer, pulse-echo, pitch-catch, peak density, ductal carcinoma in situ

## Abstract

Ultrasound is commonly used as an imaging tool in the medical sector. Compared to standard ultrasound imaging, quantitative ultrasound analysis can provide more details about a material microstructure. In this study, quantitative ultrasound analysis was conducted through computational modeling to detect various breast duct pathologies in the surgical margin tissue. Both pulse-echo and pitch-catch methods were evaluated for a high-frequency (22–41 MHz) ultrasound analysis. The computational surgical margin modeling was based on various conditions of breast ducts, such as normal duct, ductal hyperplasia, DCIS, and calcification. In each model, ultrasound pressure magnitude variation in the frequency spectrum was analyzed through peak density and mean-peak-to-valley distance (MPVD) values. Furthermore, the spectral patterns of all the margin models were compared to extract more pathology-based information. For the pitch-catch mode, only peak density provided a trend in relation to different duct pathologies. For the pulse-echo mode, only the MPVD was able to do that. From the spectral comparison, it was found that overall pressure magnitude, spectral variation, peak pressure magnitude, and corresponding frequency level provided helpful information to differentiate various pathologies in the surgical margin.

## 1. Introduction

During breast-conserving surgery (BCS), a certain amount of healthy tissue surrounding the tumor is excised along with the tumor. This surrounding normal tissue is known as margin. The margin is then evaluated for any malignant cells present in it. If cancerous cells are found, the margin is called positive, otherwise it is called negative [1,2]. Achieving a negative margin is important for cancer treatment, since a positive margin significantly increases the local recurrence rate [3]. The time-consuming margin evaluation process can lead to reoperation, which always affects the physical, psychological, and financial aspects of patients [4,5]. Therefore, an instantaneous, efficient, and cost-effective margin detection process is necessary to further increase the effectiveness of breast co-serving therapy (BCT).

There are many intraoperative technologies available that are used to reduce the rate of positive margins. During lumpectomy, technologies like wire localization, radioactive seed localization, radioguided occult lesion localization, and ultrasound guidance are used for the excision of an adequate amount of tumor tissue including the margin [6]. In addition to that, intraoperative pathologic technologies like frozen section analysis, gross histology, imprint cytology, specimen radiography, and sonography are also applied to lower the positive margin rates. These pathological techniques are comparatively time-consuming and resource-intensive and require skilled operators [7,8,9]. In frozen section analysis, artifacts (compression, freezing) could occur due to the sample preparation process [10]. Imprint cytology cannot differentiate between invasive and non-invasive carcinoma [11]. In specimen radiography, there is a possibility of benign calcification being presented as malignant calcification [12]. A different type of intraoperative device (MarginProbe) is also used for margin assessment by evaluating its electromagnetic scattering, absorbance, and reflectance properties [9]. However, it also has limitations, as it provides a high number of false-positive results, which can lead to excessive tissue excision [13].

Ultrasound analysis is mostly used as an imaging tool in the medical field due to its availability, cost-effectiveness, and safe usage (no ionizing radiation) [14]. In the area of BCS, although ultrasound-guided excision was found effective (decreasing the positive margin rate) for palpable tumors, it was not conclusively recommended for the impalpable tumors [6]. Ultrasound imaging was used intraoperatively to evaluate lesions subjected to lumpectomy [15,16,17,18], but it was not as effective as other intraoperative pathologic technologies described above [12]. In the case of ultrasound imaging, only time-domain responses are used, whereas, for quantitative ultrasound, frequency-domain responses are used. It was found that frequency-domain response values are very sensitive to microstructural changes [19].

One of the asymptotic breast carcinomas is ductal carcinoma in situ (DCIS). The growth of malignant epithelial cells inside the breast duct is known as DCIS, intraductal carcinoma, or stage-zero cancer [20,21,22]. It is a non-invasive form of carcinoma, which means that the cancerous cells are yet to spread outside of the duct or to the surrounding breast tissue. It occurs due to genetic mutations in the cells of the breast duct [22]. It develops initially at a terminal duct lobular unit and then extends into the mammary ductal lobular system [23]. In the United States, around 60,000 DCIS cases are diagnosed every year and account for 20% to 25% of all breast carcinomas [24,25,26,27]. In the literature, a wide range of DCIS visibility (8–50%) through ultrasound imaging has been reported. However, overall, ultrasound imaging is considered the least useful imaging technique to detect DCIS [28]. Therefore, quantitative ultrasound response parameters, as well as spectral ultrasound analysis need to be explored in margin analysis to detect asymptotic malignancies like DCIS.

In this study, quantitative high-frequency ultrasound analysis was conducted through finite element modeling of multiple noninvasive breast duct pathologies of the surgical margin. The analysis was done on different cancerous (positive) as well as noncancerous (negative) margin models. High-frequency ultrasounds (22–41 MHz) were used for the analysis by both pulse-echo and pitch-catch or through-transmission methods. In this study, the surgical margin models consisted of normal duct, atypical ductal hyperplasia, malignant duct (DCIS), and calcified duct (benign and malignant) models. In the case of microcalcification, all three types of calcification compositions were used for modeling, i.e., (1) Calcium oxalate (CaC_2_O_4_), (2) Calcium carbonate (CaCO_3_), and (3) Hydroxyapatite (Hap, Ca_10_(PO_4_)_6_(OH)_2_). While calcium oxalate and calcium carbonate are associated with benign tumors, HAp is associated with malignant tumors [29]. The goal was to evaluate the feasibility of quantitative ultrasound to differentiate duct pathologies inside margin without calcification as well as to identify different types of microcalcifications which eventually would indicate the margin status (positive or negative). Various quantitative ultrasound response values were utilized to evaluate the material microstructure, such as attenuation coefficient, backscatter coefficient, cepstrum, and peak density [30,31,32,33]. Stromer showed that in a comparison between quantitative ultrasound peak density and pulse amplitude, peak density was more sensitive towards material microstructure [19]. In another comparative study, among the performances of different quantitative ultrasound response values (attenuation coefficient, cepstrum, peak density), peak density was found more responsive towards soft tissue microstructure [33]. The relationship between the mechanical properties of the tissue and peak density has yet to be explored. In a previous study, it was observed that peak density was related to the amount of ultrasound scattering that occurred from the microstructure of tissue phantom [34]. In some cases, where peak density was not able to capture a microstructural change, a similar response value named MPVD was found effective [35,36]. Therefore, in this study, both peak density and MPVD were used for the ultrasound evaluation of the surgical margin model. It was found that for the through-transmission method, only peak density responded to the pathological features of the breast duct with a conclusive pattern. In contrast to that, for the pulse-echo mode, only the MPVD value provided a conclusive pattern to differentiate the pathological features of the breast duct. Furthermore, frequency-domain spectral analysis was conducted to visualize the spectrum patterns. The goal was to utilize different features of the frequency spectrum to identify the margin models. From the spectral comparison, it was found that spectral features such as overall pressure magnitude, peak pressure magnitude, and spectrum jaggedness could also provide meaningful information to differentiate various types of breast duct pathologies inside the margin.

## 2. Design of the Simulation

**Figure 1:**
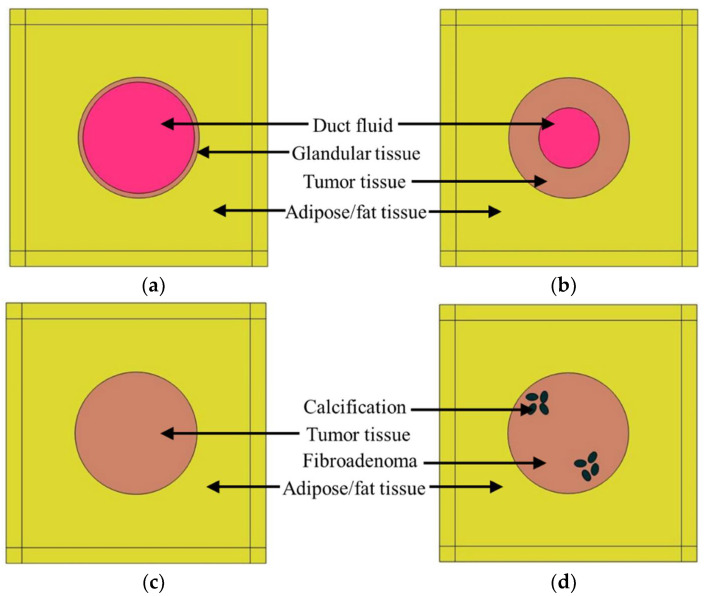
Breast duct models for the DOS, (**a**) normal duct, (**b**) ductal hyperplasia (ADH), (**c**) duct with DCIS, (**d**) duct with calcification.

The goal of this study was to explore the feasibility of quantitative ultrasound as an efficient intraoperative tool for surgical margin detection. The focus of this analysis was to identify DCIS during margin tissue evaluation. To detect DCIS during the margin tissue examination, it was necessary to differentiate it from all other noninvasive histopathological states of the breast duct. Therefore, in this study, different pathological cases of the breast duct were modeled. The first model that was considered for the study was a normal breast duct. In the simplified normal duct model shown in Figure 1a, the duct contains glandular tissue while being surrounded by fatty/adipose tissue [37]. The duct diameter varies from 1 mm to 5–8 mm starting from the lobule to the nipple [38,39]. In this case, we selected a duct diameter of 1 mm. Inside a normal duct, the glandular tissue contains two layers of epithelial cells [40]. The epithelial cell diameter is considered to be 20 µm [41]. Therefore, the glandular tissue thickness was 40 µm in this model. Duct fluid is present inside normal ducts. Based on the literature, the duct fluid was assumed to be similar to water [42]. The duct is surrounded by fatty tissue. In this 2D model, the fatty tissue dimension was 2 mm × 2 mm; this parameter was kept consistent for all the breast duct models in the design of the simulation (DOS).

Another proliferative breast disease that can be found in breast cancer tissues is ductal hyperplasia, which occurs because of the overgrowth of epithelial cells inside the duct [24]. Three types of hyperplasia can occur inside a breast duct: (1) mild hyperplasia, (2) moderate hyperplasia, and (3) atypical ductal hyperplasia (ADH). In the case of the ADH, the risk of forming DCIS is 4 to 5 times higher than for the normal duct [24]. In this study, ductal hyperplasia was modeled in the form of ADH, as shown in Figure 1b. In this case, the proliferative glandular tissue thickness was considered to be half of the duct radius i.e., 0.25 mm. The glandular tissue was modeled as a tumor or malignant tissue.

The third model of the DOS, shown in Figure 1c, was the DCIS model. Since DCIS is not invasive, the abnormal growth of epithelial cells is still confined in the duct. Therefore, in this model, the breast duct was filled with malignant/tumor tissue.

Calcification or microcalcification is a form of mineralization that can occur inside the duct due to different pathological conditions. To explore the feasibility of quantitative ultrasound in detecting calcification, the calcified duct analysis was conducted in an extended manner. All three types of micro-calcified (HAp, calcium carbonate, and calcium oxalate) ducts were included in the DOS. HAp is associated with malignancy, whereas calcium carbonate (CC) and calcium oxalate (CO) are associated with benign lesions. Two types of benign breast lumps are observed: (1) cysts and (2) fibroadenoma. The cyst is a sac filled with fluid, whereas fibroadenoma is caused by cell overgrowth inside a breast duct not associated with malignancy [43,44,45]. As a benign lesion, fibroadenoma was chosen for modeling benign calcifications (CC and CO). The duct tissue for HAp was s tumor tissue, whereas, for calcium carbonate and calcium oxalate, the duct tissue was fibroadenoma (benign lesion). Usually, clustered microcalcifications are associated with DCIS [46]. In this model shown in Figure 1d, there are two microcalcification clusters with four oval-shaped calcified structures.

To create computational variation in all surgical margin models, the duct was placed in five different positions in every margin model. In one position, the duct was placed in the center of the tissue geometry, in two positions, it was at ±0.4 mm from the center in the horizontal direction, in the remaining two positions, it was at ±0.4 mm from the center in the vertical direction.

## 3. Finite Element Analysis

During finite element analysis, acoustic propagation can be modeled either as longitudinal wave propagation or as elastic wave propagation based on the material stiffness. A material with high stiffness supports the elastic wave propagation, whereas a material with low stiffness can only accommodate the longitudinal wave propagation. In the case of tissue regions (fatty tissue, glandular tissue, tumor tissue, fibroadenoma), the stiffness is too low [37]. Therefore, inside the tissue region as well in the duct fluid, only longitudinal pressure wave propagation was considered during the analysis. The longitudinal wave propagation can be expressed through the Helmholtz equation, which is shown as Equation (1):

(1) $${𝛻}^{2}p+k^{2}p=0$$

Here, the equation expresses wave propagation in terms of a pressure field (p) inside the medium. k is the wavenumber, which is a function of sound speed (c) and frequency (f), where k=2πf/c.

In the case of the materials with microcalcification, the elastic wave propagation was supported due to the high material stiffness. The strain value of the solid structure was too small to follow a non-linear stress–strain profile because of the smaller incident pressure (≤1 Pa) in this analysis. Therefore, calcification materials are considered linearly elastic. In a linear elastic solid, the propagation of sound is expressed by particle displacement, which is governed by Navier’s equation, as shown in Equation (2):

(2) $$\rho \frac{\partial^{2}u}{\partial t^{2}}=\left(\lambda +2\mu \right)𝛻\left(𝛻.u\right)-\mu 𝛻\times \left(𝛻\times u\right)\;\;\;$$

Here, u is the displacement vector, ρ is solid density, and μ and λ are Lamé constants expressing the solid’s mechanical properties.

During finite element analysis, at the calcification material boundaries, the transfer of acoustic pressure from the tissue to the calcification and the structural acceleration to the tissue are ensured by coupling the two equations.

Mesh geometry is a very important factor in finite element analysis. In the case of an irregular or complex geometry (duct and tissue region), triangular mesh elements are effective, and in the case of a simple geometry (matched layer), a structured quadrilateral mesh can be created.

### 3.1. Simulation Physics

COMSOL Multiphysics software was used for the finite element analysis of the above-mentioned models from the DOS. COMSOL implemented both Helmholtz and Navier equations through pressure acoustics and solid mechanics physics, respectively, in the surgical margin models. In the case of the Helmholtz equation, COMSOL utilizes the pressure field p as the summation of the background pressure field $\left(p_{b}\right)$ and scattered pressure field $\left(p_{s}\right)$. The background pressure field is expressed as plane wave propagation in the $\hat{e_{x}}$ direction defined as $p_{b}=p_{0}e^{-kx}$. The scattered pressure field is the desired output in this case. Furthermore, the attenuation coefficient α of soft tissue is incorporated in the wavenumber k during the simulation, which is shown in Equation (3):

(3) $$k=\frac{2\pi f}{c}-i\ln\left(10\right)\frac{\alpha }{20}$$

Both pressure acoustics and solid mechanics physics are coupled at the microcalcification boundaries by the following boundary conditions:

(4) $$n\;.\;\left(\frac{1}{\rho }𝛻p\right)=-n\;.\;u_{tt}$$

(5) F=pn

Here, n is the normal unit vector to the boundary, $u_{tt}$ is structural acceleration, F is the acting load on solid, p is the total pressure, and ρ is the fluid density.

### 3.2. Model Description

**Figure 2:**
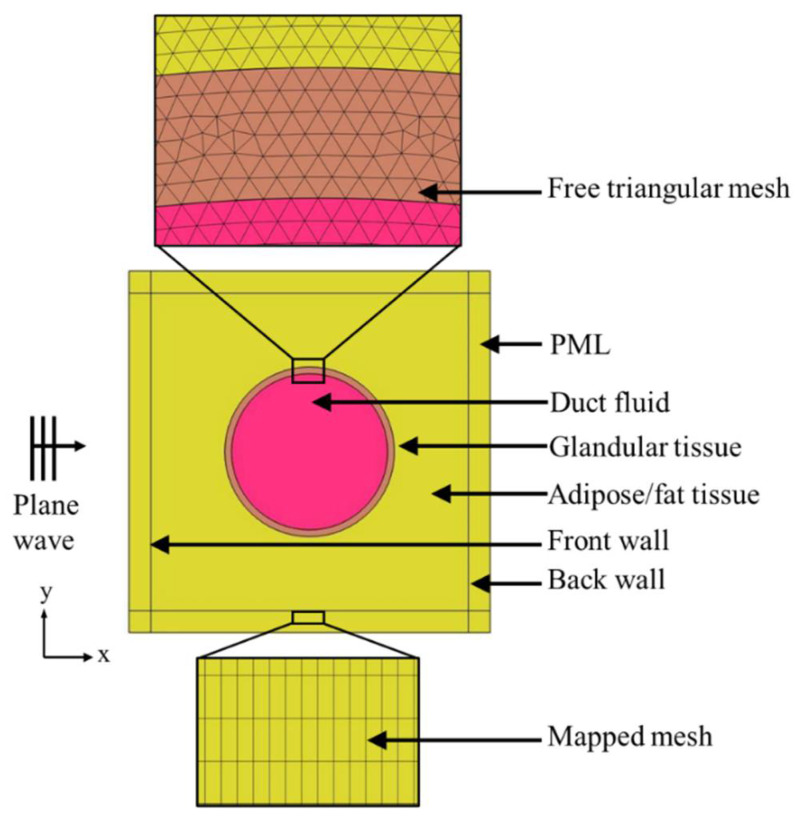
Computational model description.

Figure 2 shows the detailed model of the normal duct. Ultrasound plane waves were generated through the background pressure field in the model. The plane waves were propagating in the positive x-direction of the model geometry. A perfectly matched layer (PML) was introduced to ensure that the sound wave left the model domain without any reflection. The computational analysis was conducted for both pitch-catch and pulse-echo or through-transmission propagation modes. In the case of the experimental ultrasound analysis, a single transducer is used for the pulse-echo mode, sending and receiving the signal. In the pitch-catch or through-transmission method, two transducers are used, one transducer sending the signal, and the other transducer receiving the transmitted signal. Therefore, in this simulation, for the pitch-catch mode, after the scattered ultrasound was transmitted through the tissue, acoustic pressure was measured at the back wall. For the pulse-echo mode, after the ultrasound was reflected from the tissue, the acoustic pressure was measured at the front wall. A triangular mesh was created in the tissue geometry through the free “triangular” node, and a structured quadrilateral mesh was created in the PML region through the “mapped” node in COMSOL. A mesh sensitivity analysis was conducted to achieve convergence in the simulation results. For this analysis, the maximum element size was selected as λ/6, λ/7, λ/8, and λ/9, where λ was the wavelength (μm). Convergence was achieved in all models with a maximum element size of λ/8.

An extensive literature review was conducted to gather the material properties of different types of tissues as well as of microcalcification minerals. The material properties of all tissues, duct fluid, and microcalcification minerals are listed in Table 1 and Table 2.

**Figure 3:**
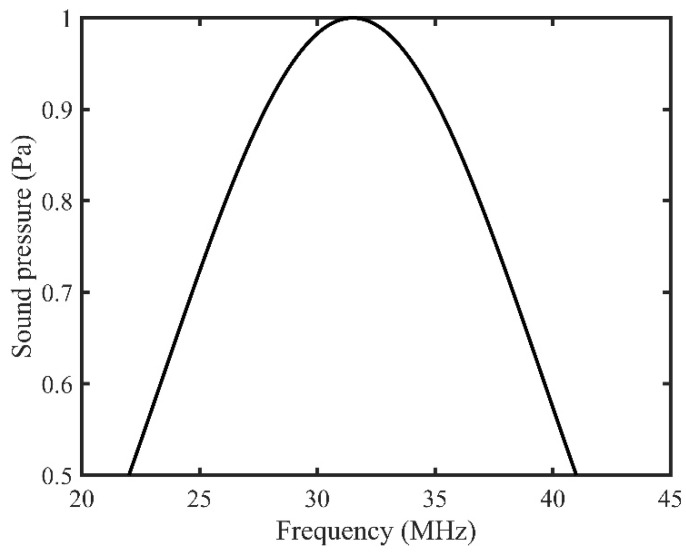
Background pressure distribution over the input frequency range.

The simulation was conducted using a high-frequency range from 22 to 41 MHz to mimic a high-frequency transducer with frequency bandwidth. In general, the ultrasound transducer sends pressure waves for a frequency range where the pressure magnitude is distributed in a bell-shaped curve and the maximum pressure magnitude is found at the center frequency. Therefore, the input background pressure for this model was distributed in a bell-shaped pattern over the frequency range, and the maximum pressure amplitude was kept at 31.5 MHz (Figure 3). Furthermore, the frequency bandwidth was selected at 50% of the maximum amplitude. Therefore, a pressure of 1 Pa was selected at 31.5 MHz, whereas at both 22 and 41 MHz, it was kept at 0.5 MPa (50% of max amplitude).

### 3.3. Response Value Measurement

The frequency step size for the simulation was selected as 100 kHz. Therefore, from 22 MHz to 41 MHz, for every 100 kHz frequency increment, the ultrasound pressure wave was sent through the simulation model geometry. The absolute value of the average scattered pressure was measured at both the front and the back wall for all 190 different frequencies. These absolute pressure values were accumulated to create the frequency spectrum in both pulse-echo and pitch-catch modes.

To calculate the peak density of the frequency spectrum, the total number of peaks and valleys were counted in the spectrum [19]. To calculate the MPVD value, first, the pressure magnitude difference between each adjacent peak and valley was measured in the frequency spectrum. Then, all those values were averaged to obtain the MPVD value for that corresponding spectrum. In the case of both peak density and MPVD, mean value and standard deviation were calculated from the data acquired from five different duct positions of each type of margin model. Additionally, the frequency spectra from all the breast duct models were compared for both pulse-echo and pitch-catch modes to extract further pathology-based information.

## 4. Results

**Figure 4:**
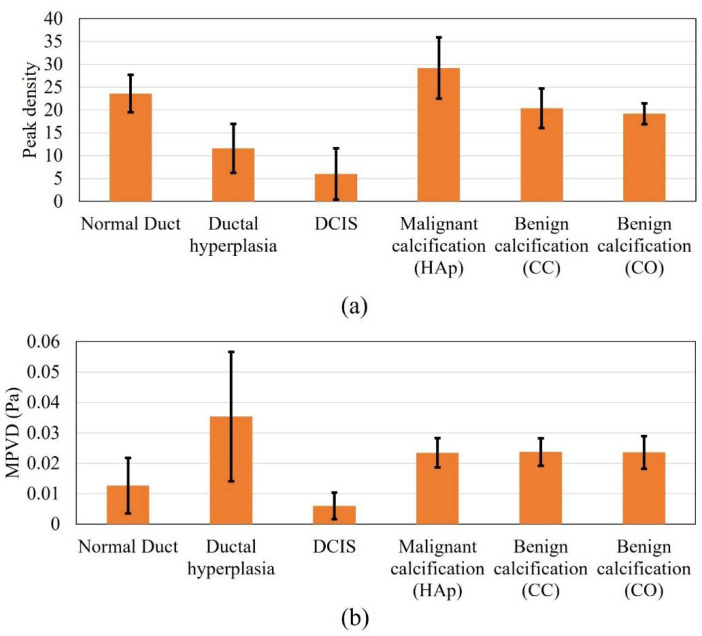
Response values of all the analyzed models in pitch-catch mode: (**a**) peak density and (**b**) MPVD.

Figure 4 shows the peak density and MPVD data from all the computational margin models for the pitch-catch ultrasound propagation mode. The mean peak density result was high for the normal duct inside the margin. With the progression to carcinoma i.e., for hyperplasia and DCIS, the mean peak density started to decrease. When the calcification was introduced in the duct, the peak density value increased again compared to the other stages of carcinoma progression. For the calcification models, from the peak density results, it was observed that both benign calcifications (CC and CO) had a similar mean peak density, which was lower than that for the malignant calcification (HAp). Without calcification, the standard deviation range of cases with the malignant margin (ductal hyperplasia and DCIS) was outside (lower) the standard deviation range of the normal margin (normal duct). The standard deviation range of the calcified margin models was inside the standard deviation range of the normal margin at some level.

In the case of the margin of the normal duct, three different types of material, i.e., fatty tissue, glandular tissue, and duct fluid, were present. Thus, multiple ultrasound scattering occurred (at the fat–glandular tissue and glandular tissue–duct fluid interfaces). Therefore, the peak density value was high, because peak density increases with an increasing level of scattering [34]. In the case of ductal hyperplasia inside the margin, although it contained similar materials as the normal duct, the duct fluid region decreased to almost half, and the glandular tissue was substituted by tumor tissue, which covered more than half of the duct area. Compared to the duct fluid, tumor tissue possessed a very high attenuation coefficient. Therefore, the overall attenuation coefficient of the tumor–fluid region was higher than that of the glandular–fluid region from the normal duct. Accordingly, the acoustic scattering of the tumor–fluid interface became very insignificant, since acoustic intensity decreased more compared to the normal duct. Therefore, the hyperplasia model provided a lower peak density compared to the normal duct model. In the case of the pure DCIS inside margin, there were only two materials (fatty tissue and tumor tissue). Therefore, the scattering level was lower than in the previous two models and yielded a further lower peak density value. When calcification was added to the model, multiple scattering again started to occur. Therefore, the scattering level was high compared to the previous two carcinoma models. While comparing the benign and malignant calcification results, the stiffness of the CC and CO was much higher compared to that of HAp. From the literature, it was found that with increased material stiffness, the peak density decreases, which complements our results [59].

In the case of the MPVD results, the mean MPVD value of the hyperplasia model was the highest, with the maximum standard deviation. The mean MPVD value for the normal duct and DCIS models was low compared to those of the hyperplasia and calcification models. Overall, the MPVD data failed to establish a meaningful trend in relation to different duct pathologies. It was observed that both benign calcifications had a similar MPVD value, which was slightly higher than the malignant calcification value.

**Figure 5:**
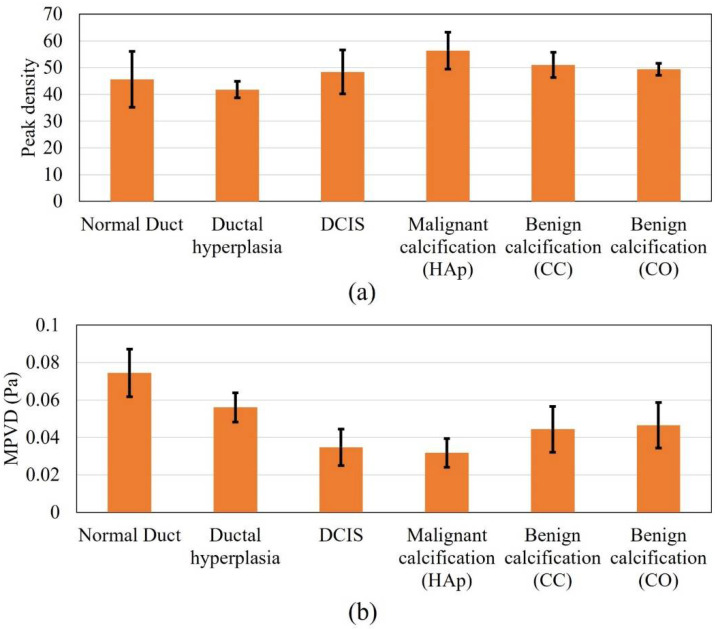
Response values for all the models in pulse-echo mode, (**a**) peak density and (**b**) MPVD.

Figure 5 shows the results for all the DOS models for the pulse-echo ultrasound propagation mode. In the case of the peak density results, all the margin models presented higher peak density values, except for ductal hyperplasia. The highest mean value was recorded for the malignant calcification model. Overall, the peak density result of the pulse-echo mode failed to establish a meaningful trend in relation to the margin models. In the case of the pulse-echo mode, the scattered wave interacted with the incident wave while reaching the front wall of the model geometry. This might be one reason for the inconclusive relationship.

In the case of the MPVD data, the result was meaningful and was similar to the peak density result of the pitch-catch method. The mean MPVD of the normal duct was the highest and, progressing to carcinoma, started to decrease. However, when calcification was added to the model, the MPVD started to increase again. In contrast to the peak density pattern from the pitch-catch method, the malignant calcification MPVD value was lower than the benign calcification MPVD value. Still, both benign calcification models provided similar MPVD values, as observed for the pitch-catch peak density (Figure 5a). Without calcification, the standard deviation range of the malignant margin cases (ductal hyperplasia and DCIS) was outside (lower) of the standard deviation range of the normal margin (normal duct). The standard deviation range of the calcified margin models was also outside (lower) of the standard deviation range of the normal margin. The difference in the ranges (malignant vs. benign calcification) was partly distinguishable. From previous research, it was found that when the peak density results provide similar values for different models, MPVD becomes effective [36]. By definition, MPVD depicts the average peak-to-valley pressure magnitude difference in a frequency spectrum. Therefore, even though the peak density was similar for all models, the spectrum patterns differed from each other because of the structural difference between the models. Accordingly, the different pressure magnitudes of all the peaks and valleys from different spectra provided a pattern in the MPVD data.

In addition to analyzing the peak density and MPVD results, the frequency spectrums of all models were compared directly for both pulse-echo and pitch-catch ultrasound analysis modes. The goal of this analysis was to find specific patterns in the frequency spectrum which could provide more information about the different breast duct pathologies.

**Figure 6:**
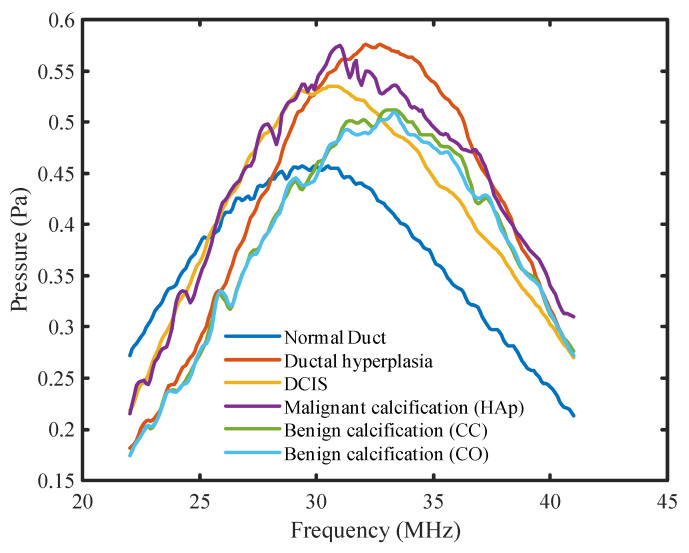
Frequency spectrums for all DOS models for the pitch-catch mode.

Figure 6 shows the frequency spectrums of all the margin models for the pitch-catch method of ultrasound analysis. From the figure, it was observed that the peak pressure values for all the spectrums were at different frequency levels. The DCIS peak pressure was shifted to the right by approximately 3 MHz compared to the normal duct peak pressure. In the case of ductal hyperplasia, the peak pressure was shifted further right by an additional 3 MHz compared to the DCIS model. Furthermore, only the peak pressure for the normal duct model occurred before the center frequency. In the case of DCIS and malignant calcification, the peak pressure was approximately at the center frequency. For the rest of the model spectrums, the peak pressure occurred after the center frequency. When calcification was added to the model, a larger pressure magnitude variation (jaggedness) was observed compared to non-calcified models. The benign calcification (CC and CO) spectrums reached their peak pressure after 4 MHz compared with the malignant calcification (HAp) spectrum. The shifting peak pressure along the frequency axis was an outcome of the resonant frequency of the material. At the resonant frequency, the material generates maximum sound pressure when subjected to external acoustic vibration. The resonant frequency changes with the material’s properties. Therefore, with various tissue materials in the breast duct, different surgical margin models possessed different resonant frequencies. Accordingly, the peak pressure of different models occurred at different frequencies. Thus, the frequency corresponding to the peak pressure could be an indicator of a material’s properties as well as of breast tissue pathologies. Additionally, the benign calcification spectrums were similar to each other, explaining why they had similar peak density and MPVD. Overall, there was a clear difference between the malignant and benign calcification spectrums.

**Figure 7:**
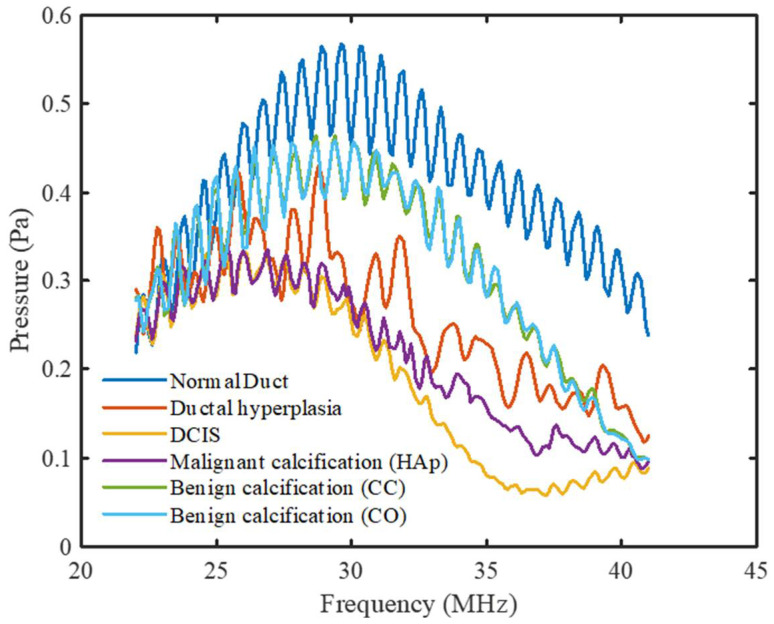
Frequency spectrums for all DOS models for the pulse-echo mode.

Figure 7 shows the frequency spectrums of all the breast duct models for the pulse-echo method of ultrasound analysis. Periodicity was observed in the normal duct spectrum due to the small thickness of the duct. The reflected acoustic wave from the duct wall came back to the front wall during its compression (high pressure) and rarefaction (low pressure) stages at the corresponding frequencies. With added malignancy and calcification in the normal duct geometry, the reflected wave from the tumor tissue and calcification minerals started to contribute to the previous spectrum with different phases. Therefore, the periodicity in the spectrum started to fade away with malignancy. The overall patterns of the pulse-echo spectrums showed an opposite trend compared to that of the pitch-catch spectrums. The peak pressure magnitude of the spectrums shifted gradually to the left with increased calcification level. Furthermore, the normal duct spectrum, in this case, had the highest overall pressure magnitude level, with larger pressure magnitude variations between the peaks and the valleys. Both the pressure level and the peak-to-valley magnitude variation started to decrease with the progression to carcinoma. With the addition of calcification, they started to increase again. The calcification spectrums also showed an opposite trend compared to the previous method. In this case, the malignant calcification spectrum had a lower level of pressure magnitude and a smaller variation compared to the benign calcification spectrums. However, similar to the previous mode, the CC and CO (benign) spectrum patterns were very similar as well as distinguishable from the HAp (malignant) spectrum.

## 5. Discussion

For non-calcified models, peak density of quantitative ultrasound gradually decreased with a developing malignancy inside the breast duct of surgical margin during pitch-catch ultrasound analysis. A similar response was observed for the MPVD data with the pulse-echo method. The standard deviation range in both methods indicated that there was a possibility of misdiagnosis when detecting malignancy in the margin. Therefore, experimental validation is required for these techniques to evaluate their performances in terms of sensitivity and specificity.

The malignant calcification peak density was higher than the benign calcification peak density in the pitch-catch mode, whereas, for the pulse-echo mode, the opposite result was found for the MPVD value. In both cases, the response values for the two benign calcifications (CC and CO) were very close. Since the properties of fibroadenoma and tumor tissue were very similar, the drastic change in calcification mineral properties (sound speed and stiffness) mostly contributed to the variation of the response values.

Regarding spectral comparison, some features of the frequency spectrums were found useful to differentiate the spectrums, i.e., overall pressure magnitude level, peak pressure magnitude, and jaggedness level of the spectrum. In the pulse-echo mode, DCIS can be identified by the low MPVD value as well as the low-pressure magnitude level in the spectrum with irregular jaggedness. Malignant calcification can be identified by a similar spectrum pattern with more jaggedness. In the pitch-catch mode, a lower peak density value can indicate the presence of DCIS. Malignant calcification can be identified by a high peak density and high-pressure level in the frequency spectrum, with distinguished and irregular jaggedness. By using quantitative ultrasound, benign and malignant calcifications were differentiated at multiple levels. Therefore, the authors’ understanding is that quantitative ultrasound analysis can be used as an effective tool to identify different pathology levels of the margin intraoperatively. This characterization method can be implemented during breast-conserving surgery to evaluate the surgical margin. Since the results can be obtained instantaneously, the surgeons will be able to understand the nature of the surgical margin histology (benign, malignant, or calcified) shortly after tumor and margin excision. This will help surgeons to decide whether further excision is necessary during the surgery. It is expected that combining this method of margin characterization with conventional ultrasound imaging will increase the efficiency of intraoperative ultrasound analysis by reducing the reoperation rate among breast cancer patients. Furthermore, both pulse-echo and pitch-catch methods were found effective for this application although, for the latter method, it might be complicated to implement it at the experimental level.

## 6. Conclusions

In this study, computational ultrasound analysis was conducted on different surgical margin models that were created based on various malignant and benign breast tissue conditions. Quantitative response parameters of high-frequency ultrasound (22–41 MHz) were used for the analysis. The analysis was conducted in both pulse-echo and pitch-catch ultrasound modes. It was found that peak density in the pitch-catch method and MPVD in the pulse-echo method had the potential to identify different stages of ductal carcinoma in the surgical margin. In both cases, the response values showed a pattern allowing the differentiation of various breast duct pathologies inside the margin. The analysis was further extended to compare the frequency spectrums of all models. Spectral features like overall pressure magnitude, peak pressure magnitude, spectrum jaggedness were observed during the comparison. The pulse-echo and pitch-catch modes showed opposite traits for the spectrums in terms of these features. Compared to the pitch-catch mode, the pulse-echo mode provided a more conclusive pattern in all the spectrums. Overall, quantitative ultrasound analysis was found effective for both methods to differentiate pathological duct features. Along with ultrasound imaging, this analysis can be introduced to establish ultrasound as the intraoperative margin analysis tool.

## Figures and Tables

**Table 1:** Material properties of all tissues and duct fluid.

	Density (ρ)	Sound Speed (c)	Attenuation Coefficient (α)
	$kg/m^{3}$	m/s	Np/m−MHz
Fatty tissue	869 [47]	1422 [48]	5.7 [47]
Glandular tissue	874 [47]	1487 [48]	10.5 [47]
Tumor tissue	1041 [49]	1548 [48]	11.28 [31]
Fibroadenoma	1060 [50]	1520 [51]	10.82 [31]
Duct fluid (water)	1000 [52]	1480 [52]	0.025 [52]

**Table 2:** Material properties of the microcalcification minerals.

	Density (ρ)	Sound Speed (c)	Young’s Modulus	Poisson’s Ratio
	$kg/m^{3}$	m/s	GPa	
Hydroxyapatite (HAp)	3180 [29]	1374 [29,53]	6 [53,54,55,56]	0.27 [55]
Calcium Carbonate	2930 [29]	5486 [29,57]	88.19 [57]	0.32 [57]
Calcium Oxalate	2200 [29]	4785 [29,58]	50.38 [58]	0.32 [58]

## Data Availability

Data available upon request to the authors.
